# Prevalence and Pattern of Tobacco‐Related Habits Among Dental Students: A Cross‐Sectional Questionnaire Survey

**DOI:** 10.1155/ijod/4347171

**Published:** 2026-07-26

**Authors:** Deepak Kumar Singhal, Vathsala Patil, Nishu Singla, Ramprasad Vasthare Prabhakar, Chetana Chandrashekar, Jeevan V. Sambasivan

**Affiliations:** ^1^ Department of Public Health Dentistry, Manipal College of Dental Sciences, Manipal Academy of Higher Education, Manipal 576104, India, manipal.edu; ^2^ Department of Oral Medicine and Radiology, Manipal College of Dental Sciences, Manipal Academy of Higher Education, Manipal 576104, India, manipal.edu; ^3^ Department of Oral and Maxillofacial Pathology and Oral Microbiology, Manipal College of Dental Sciences, Manipal Academy of Higher Education, Manipal 576104, India, manipal.edu

**Keywords:** dental students, India, prevalence, SDG goals, smoking, tobacco

## Abstract

**Background:**

Tobacco consumption is one of the leading causes of preventable death globally, responsible for over 8 million deaths annually according to the World Health Organization (WHO) Global Tobacco Epidemic Report 2023. Dental professionals are instrumental in eliminating this problem. However, their own tobacco habit may prevent them from offering their patients counselling and guidance on quitting. In order to assess the prevalence and pattern of tobacco‐related habits among dental students, a survey was conducted.

**Materials and Methods:**

A cross‐sectional survey was undertaken among 370 undergraduate students enrolled in a dental college in Southern India. Data collection was carried out using a structured, close‐ended questionnaire designed to assess tobacco usage patterns, types of tobacco products consumed, as well as participants’ knowledge, attitudes and behaviours pertaining to tobacco cessation. The collected data were subjected to descriptive statistical analysis using statistical package for the social sciences (SPSS) Version 23.0, with a *p*‐value of less than 0.05 considered indicative of statistical significance.

**Results:**

Out of 370 students, 8.6% were current smokers, 23.2% were past smokers and 68.2% had never smoked. A total of 1.1% were current tobacco chewers and 2.7% were past tobacco chewers. Majority are cigarette smokers. A significantly high number (79.9%) of past users smoked for less than 2 years as compared to 59.4% of current users who smoke for more than 2 years. A total of 53.2% of current users never tried to quit, and 25% don’t want to quit the habit.

**Conclusion:**

Dental professionals who smoke cigarettes themselves may experience diminished professional credibility as promoters of healthy behaviour, which could, in turn, compromise the persuasiveness and efficacy of their tobacco cessation counselling interventions. It may have detrimental effects on society if they themselves are involved in tobacco consumption practices. Enforcement of policies should be followed strictly at all levels to prevent tobacco usage among students. Quitting tobacco is a cornerstone of public health efforts and is strongly linked to global goals of sustainable good health and well‐being (Sustainable Development Goal 3 [SDG3]), because it leads to immediate and long‐term health improvements, reducing the burden of disease over a person’s lifetime.

## 1. Introduction

Tobacco use continues to be one of the greatest hazards to public health, with over 8 million tobacco‐related deaths annually, and tobacco control is still a top concern for global health. Low‐ and middle‐income nations account for the majority of these fatalities. Every year, 1.3 million nonsmokers die from secondhand tobacco smoke [1].

In India, tobacco usage is a leading cause of illness and mortality. Tobacco smoking is one of the main risk factors for NCDs (non‐communicable diseases), which account for over 53% of all fatalities in India. A total of 35% of all individuals over the age of 15 use tobacco, according to the Global Adult Tobacco Survey, India, 2016–17. India is the third‐largest producer and second‐largest user of tobacco, and a wide range of tobacco products are offered at extremely low costs [2].

The World Health Organization (WHO) Framework Convention on Tobacco Control (FCTC) emphasises the significance of the role that physicians, dentists, nurses, pharmacists, optometrists and others play in helping tobacco users quit and avoid tobacco use by offering them brief counselling or even straightforward advice [3]. Importantly, the initiation of tobacco use tends to occur early between the ages of 14 and 25. This is a crucial period of life during which preventative interventions and awareness programs can greatly shape an individual’s health trajectory [4]. University campuses provide a key venue for tobacco control, given the particular sociocultural, academic and life circumstances that characterise this period of the students’ lives. Dentists play a crucial role in that they are the earliest to observe the manifestations of tobacco in the oral cavity, such as tobacco staining of teeth and mucosa, oral halitosis and development of precancerous and cancerous lesions. As part of a health worker’s role, dental practitioners have a role to play in educating the public about oral health and health‐promoting lifestyle habits. This includes raising awareness about the negative health consequences of tobacco and assisting patients to quit tobacco use [5].

Teaching dentistry students how to stop smoking may have an effect on their future careers by assisting smokers through interviews, straightforward counsel, or referrals to clinics [6–8]. Health professional students who get cessation training have the potential to make a substantial contribution to tobacco reduction initiatives [8]. However, health professionals who themselves smoke may hesitate from providing cessation advice and counselling to their patients as it may be harder to effectively encourage patients to quit [6, 7].

Thus, it’s critical to comprehend the variables influencing tobacco use among dentistry students and whether they recognise tobacco use as a public health issue. Therefore, the purpose of this study was to assess the prevalence of tobacco usage among dental students in a college in South India and to assess the pattern of tobacco usage, nicotine dependency and cessation attempts among dental students.

While tobacco use among dental students in India has been previously investigated, the present study addresses an important gap in the literature. Most earlier studies were conducted more than a decade ago, and contemporary data on tobacco use prevalence among the current generation of dental students, particularly future oral health professionals in Coastal Karnataka, remain limited. In addition, this study uniquely integrates the assessment of tobacco use patterns, nicotine dependence using the Fagerström Test for Nicotine Dependence (FTND) scale, and cessation‐related behaviours within a single institutional cohort, thereby providing a comprehensive and updated profile of tobacco habits among undergraduate dental students in Coastal Karnataka, India. Considering the evolving tobacco product landscape, including the increasing use of hookah and combination tobacco products observed in the present study, updated surveillance data are essential for the development of targeted tobacco cessation interventions within dental curricula.

## 2. Materials and Methods

This cross‐sectional survey was conducted among undergraduate dental students at a dental college in South India to assess tobacco use patterns, nicotine dependence and awareness regarding its harmful effects. The study protocol was submitted to the Kasturba Medical College and Kasturba Hospital Institutional Ethics Committee, and approval was obtained (IEC 179/2018). Informed written consent was obtained from all participants prior to data collection. Prior to the commencement of the survey, official administrative permission was obtained from the head of the institution. A total of 494 undergraduate students of all academic years were invited to participate in this survey. A convenience sampling method was employed, wherein all students present on the three survey dates (Jan–March 2019) and who provided informed written consent were included in the study, resulting in a final sample of 370 participants, representing ~75% of the eligible student population. Students who did not provide informed written consent were not allowed to participate in the study.

**Figure 1:**
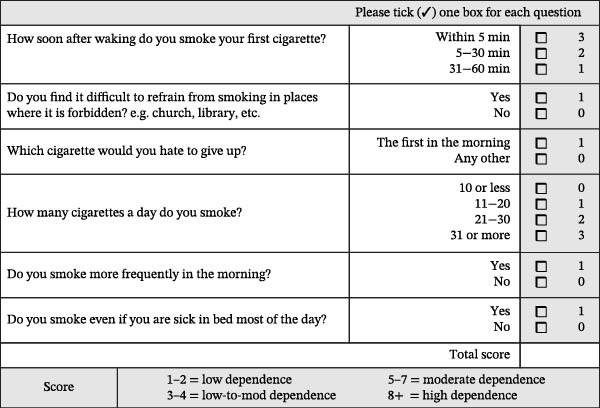
Fagerström Test for Nicotine Dependence.

Data collection was carried out using a structured close‐ended questionnaire to gather information about their demographics, tobacco usage, type and pattern of tobacco product use. Nicotine dependence was assessed using the ‘Fagerström Test for Nicotine Dependence’. Their awareness about the harmful effects of tobacco and previous tobacco cessation attempts by them were also collected. The FTND questionnaire, is a validated, widely used instrument comprising a 6‐item scale [9] to assess the degree of nicotine dependence. The score for the scale ranges between 0 and 10, with increasing value indicating increasing level of addiction (Figure 1). All questions in the questionnaire were pilot‐tested for content validity prior to the main survey and answered by the participants. A pilot study was conducted among 20 dental students, who were not included in the main study, to assess the usability, appropriateness and acceptability of the questionnaire. Cronbach’s alpha coefficient was 0.89, demonstrating the good reliability of the questionnaire used for this study.

For this study, tobacco users were categorised as follows: current smokers—participants who reported active use of any smoked tobacco product at the time of the survey; past smokers—participants who had previously used smoked tobacco products but had ceased at the time of the survey and never smokers—participants who had never used any smoked tobacco product. Similarly, current tobacco chewers referred to participants actively using smokeless tobacco products, and past chewers referred to those who had previously used such products. The term ‘current users’ in Tables 1 and 2 refers collectively to all participants currently using any form of tobacco product, whether smoked or smokeless. All those who are chewing tobacco currently are also current smokers.

**Table 1:** Quitting and cessation activities: for current users.

Parameters	*N* (%)
Have you ever tried to quit smoking/chewing tobacco?	
Yes	12 (46.8%)
No	17 (53.2%)
Do you want to quit smoking/chewing tobacco?	
Yes	24 (75%)
No	8 (25%)
If yes, why do you want to quit (factors responsible for cessation activities)?	
Awareness of health hazard	18 (75%)
It is a bad habit	6 (25%)
Presence of a medical condition	0
Combination	0
Have you ever received help or advice from professionals to stop using tobacco?	
Yes	5 (15.6%)
No	27 (84.4%)

**Table 2:** Quitting and cessation activities: for past users.

Parameters	*N* (%)
When did you quit smoking/chewing tobacco?	
In last 1 year	50 (58.1%)
More than 1 year ago	36 (41.2%)
Why did you quit smoking/chewing tobacco?	
Health reasons	6 (6.9%)
Awareness about harmful effects	64 (74.4%)
Financial reasons	2 (2.3%)
Miscellaneous	14 (16.2%)
Did you take help or advice from professionals to stop using tobacco?	
Yes	2 (2.3%)
No	84 (97.7%)

The statistical package for the social sciences (SPSS) Version 23.0 was used for data analysis. Descriptive statistics, including means and percentages, were computed to summarise demographic variables of study participants. Inferential analysis of categorical variables was done with the chi‐square test of significance. Results with a *p*‐value < 0.05 were considered statistically significant.

## 3. Results

A total of 370 study participants were recruited, including 27% males and 73% females. The mean (SD) age of the participants was 21.05 (1.82) years. Most participants lived in university hostels (*n* = 286; 77.3%). Of the 370 study participants, 32 (8.6%) were found to be current smokers and 86 (23.2%) ex‐smokers. A total of 252 (68.2%) study participants had never smoked before. Of the 370 study participants, 1.1% of participants were currently using tobacco chewing products and 2.7% were ex‐users. A total of 96.2% of the participants had never used tobacco chewing products (Table 3). Among current smokers, 78.1% used cigarettes, whereas among ex‐smokers, 50% used cigarettes and 36.1% used a combination of all smoking products. The difference in the usage of cigarettes between current and ex‐smokers was found to be statistically significant (*p* = 0.014).

**Table 3:** Socio‐demographic characteristics and tobacco usage status of study participants.

Parameters	*N* (%)
Gender	
Males	100 (27%)
Females	270 (73%)
Age	
Mean (SD) in years	21.05 ± 1.82
Residence	
Hostel	286 (77.3%)
Outside hostel	84 (22.7%)
Smoking status	
Current smokers	32 (8.6%)
Past smokers	86 (23.2%)
Non‐smokers	252 (68.2%)
Chewing status	
Current chewers	4 (1.1%)
Past chewers	10 (2.7%)
Non‐chewers	356 (96.2%)

Approximately two‐thirds of the study participants smoked fewer than two cigarettes per day. A significant difference was also observed between current and past users with respect to the duration of smoking (*p* < 0.001). Among past users, 79.9% had smoked for less than 2 years, whereas 59.4% of current users reported a smoking duration of more than 2 years (Table 4).

**Table 4:** Pattern of tobacco‐related habits among the study participants.

Parameters	Current users *N* (%)	Past users *N* (%)	*p*‐Value
What do you smoke?			
Cigarette	25 (78.1%)	43 (50%)	0.014
Hookah	3 (9.37%)	12 (13.9%)	
Combinations	4 (12.5%)	31 (36.1%)	
Smoking frequency			
Daily	10 (31.2%)	13 (15.11%)	0.6
Occasionally	22 (68.8%)	73 (84.8%)	
Number of cigarette per day			
Less than 2/day	20 (62.5%)	59 (68.6%)	0.75
2 or more/day	12 (37.5%)	27 (31.4%)	
Number of pack per day			
Less than 1 pack/day	32 (100%)	86 (100)	–
More than 1 pack/day	0	0	
Smoking duration in years			
Less than 2 years	13 (40.6%)	68 (79.9%)	0.001
More than 2 years	19 (59.4%)	18 (20.1%)	

*Note:* *p* < 0.05 is considered as statistically significant. Chi‐square test of significance for analysis.

Table 5 shows the level of dependency using the FTND scale among current users of tobacco products. Among them, 59.4% demonstrated low dependency, 28.1% had low‐to‐moderate dependency and 12.5% exhibited moderate dependency on tobacco products.

**Table 5:** Nicotine dependency of current users on tobacco products.

Parameters	*N* (%)
FTND dependency level	
Low (1–2)	19 (59.4%)
Low‐to‐moderate (3–4)	9 (28.1%)
Moderate (5–7)	4 (12.5%)
High (8 and above)	0 (0.0%)

*Note:* Standard scoring: 0–2 = very low dependence; 3–4 = low dependence; 5–6 = medium dependence; 7–8 = high dependence; 9–10 = very high dependence. For the purpose of this study, scores were grouped as low (1–2), low‐to‐moderate (3–4) and moderate (5–7). No participant scored in the high or very high range (score ≥8).

Abbreviation: FTND, Fagerström Test for Nicotine Dependence.

Most participants were aware of the harmful effects of tobacco, not only in relation to oral and lung cancer but also its association with other systemic illnesses like hypertension, stroke, asthma, and emphysema (Table 6). The mean (SD) age of initiation of tobacco use was 18.81 (2.1) years, and 31.36% of users reported initiating the habit more than 2 years ago. The primary reasons for initiating tobacco use were curiosity and recreation (44.9%), followed by peer pressure (29.6%). Academic stress and personal problems were identified as the main reasons for the continuation of the habit.

**Table 6:** Awareness about harmful effects of tobacco and factors responsible for initiation.

Parameters	*N* (%)
Do you know that chronic use of tobacco can cause oral and lung cancer?	
Oral cancer only	0
Lung cancer only	0
Oral and lung cancer	370 (100%)
Neither	0
Do you know that tobacco contributes to hypertension/stroke/asthma/emphysema?	
Yes	368 (99.5%)
No	2 (0.5%)
Do you notice the anti‐tobacco warnings written on tobacco products?	
Yes	368 (99.5%)
No	2 (0.5%)
At what age did you start using tobacco?	
Mean age of initiation (in years)	18.81(2.1)
How many years ago did you first started using the product?	
Less than 2 years ago	81(68.64%)
More than 2 years ago	37 (31.36%)
Why did you start using tobacco?	
Curiosity and fun/showoff	53 (44.9%)
Peer pressure	35 (29.6%)
Work/academic stress	5 (4.24%)
Personal problems	5 (4.24%)
Other factors	20 (16.91%)
Why are you continuing using tobacco?	
Out of habit	4 (12.5%)
Peer pressure	2 (6.2%)
Work/academic stress	6 (18.75%)
Personal problems	12 (37.5%)
Other factors	8 (25.0%)

Table 1 shows that 53.2% of current users had never attempted to quit, and 25% reported no intention to quit the habit. Only five current users had received professional help or advice for tobacco cessation. Among past users, 58.1% had quit the habit within the last year, primarily due to awareness of its harmful effects (Table 2). Most of the past users (97.7%) reported that they did not seek or receive professional help or advice to stop using tobacco.

## 4. Discussion

Our country is facing a growing tobacco epidemic. Health care providers are crucial to tobacco control and cessation, both for the individual patient and for society at large. Dental students, being future dental professionals, are the first ones to examine the oral cavity of patients [10]. Therefore, it is the primary responsibility of these oral health care students to assist smokers in quitting and to stop others from engaging in smoking‐related behaviours [11].

The study findings revealed that among the 370 students, 32 (8.6%) were current smokers, 86 (23.2%) were former smokers, and 252 (68.2%) had never smoked. Regarding tobacco chewing, 1.1% of participants were current users, 2.7% were former users, and the vast majority (96.2%) had never chewed tobacco. According to a 2005 WHO survey, almost 10 (9.6%) of third‐year dentistry students in India presently smoke cigarettes, and 3.7% currently use other tobacco products [12]. Rajasundaram et al. [13] obtained similar results. High prevalence of tobacco usage was reported among dental students at King Saud University [14] and dental students at Davangere [15]. These figures are broadly consistent with the 10.9% ever‐tried and 4.8% current smoking prevalence reported among medical students in Chennai by Boopathirajan and Muthunarayanan [16], suggesting that tobacco use rates among health professional students in India cluster within a similar range irrespective of discipline.

Almost all the study subjects were aware that consumption of tobacco and tobacco‐related products is injurious to not only oral health but also to general health. These findings are in concordance with the study done by Kumari et al. [17]. This could be one of the possible reasons for the low prevalence of tobacco usage among the present study participants, and those having the habit could be motivated to quit smoking. Comparable levels of awareness were documented in medical students, where nearly all respondents recognised tobacco as a risk factor for systemic illness [16], reinforcing the notion that high awareness does not necessarily translate into cessation behaviour among health professional students.

Dentistry has a reputation as a demanding, stressful, technically exacting and sometimes personally hazardous career [18, 19]. The major reasons for the initiation of tobacco usage were curiosity and fun and peer pressure. The reasons for the continuation of consumption of tobacco and tobacco‐related products were also assessed. The following reasons reported by current users are academic stress, personal problems and peer influence. Given that students utilise tobacco smoking as a coping mechanism for stress, this research should be taken extremely seriously [20]. Similar findings were reported by AlSwuailem et al. [14], Kumari et al. [17], and Chitalkar et al. [21].

The findings of this survey show that 46.8% of current users attempted to quit, and 75% reported an intention to quit the habit. This means that the majority of smokers will give up provided they receive appropriate follow‐up at regular intervals and adequate nicotine cessation therapy. This finding is similar to that reported by Kothari [22]. Importantly, among medical students surveyed by Boopathirajan and Muthunarayanan, 34.6% had tried to stop smoking in the past year, with the majority citing a willingness to quit [16], a finding convergent with the present study and indicative of a receptive target population for structured cessation interventions. Notably, in the present study, 53.2% of current users have never tried to quit and 25% do not want to quit the habit. Since the dental students themselves are indulged in the smoking habit, they need tobacco cessation counselling by the professionals. This reflects an alarming situation and demands tobacco cessation measures to be adopted by these students, who happen to be health promoters and role models for society.

The most concerning observation from both studies was the lack of formal training in tobacco cessation. In the present study, a few current users have received professional help/advice for tobacco cessation. In the study carried out by Boopathirajan and Muthunarayanan on medical students, 23.6% of students have been given formal training in smoking cessation techniques, which reiterates the deficiency of the present health professional curriculum. Similarly, 70.6% of medical students in the study [16] felt that health professionals who smoke may be less likely to recommend patients to stop smoking, similar to the observation in the present study that dental students who use tobacco are less likely to recommend smoking cessation to their patients, thereby emphasising the need for formal training in tobacco cessation in undergraduate health professional curricula.

The results of the present study are comparatively better than those reported among dental students in Patna city, where none of the subjects made any attempt to quit tobacco [17]. Additionally, the process of conducting such surveys may contribute to the dissemination of knowledge and enhance awareness about the ill effects of tobacco, thus serving as an indirect benefit of the study.

One of the limitations of the study would be reporting bias, as students would hesitate to accept the habit of smoking. The single‐institution design of this study significantly limits the generalizability of the findings. The results reflect the tobacco use patterns of undergraduate dental students at one institution in South India and should not be extrapolated to represent Indian dental students as a whole, given the considerable variation in institutional culture, geographic location, student demographics and campus policies across dental colleges in India. Multi‐institutional studies with stratified sampling across different regions of India are warranted to establish nationally representative prevalence estimates.

Another important limitation is the potential for a social desirability bias. Participants, being dental students with an awareness of the negative health consequences of tobacco, may have underreported their tobacco use to appear consistent with their professional identity. This could result in an underestimate of the true prevalence of tobacco use in this population. Future studies may consider using validated anonymous digital platforms or biochemical verification, such as a carbon monoxide breath analyser and salivary cotinine estimation, to mitigate this bias.

The comparatively lower prevalence of tobacco use observed in the present study may be attributed to several contextual factors. First, the institution is located in a university town where tobacco‐free campus policies are actively enforced, which may deter tobacco initiation or continuation among students. Second, the predominantly female composition of the study sample (73%) likely contributes to the lower overall prevalence, as tobacco use rates are consistently lower among women in India, consistent with national data from the Global Adult Tobacco Survey 2016–17. Third, the possibility of underreporting due to the social desirability bias, as discussed above, cannot be excluded. Finally, the single‐institution design of the study may reflect institution‐specific cultural, academic, or policy‐driven norms that reduce tobacco uptake, thereby limiting the generalizability of the findings to other dental institutions across India.

The findings of this study have broader implications for global public health goals. Tobacco cessation is directly aligned with the United Nations Sustainable Development Goal 3 (SDG3), which aims to ensure healthy lives and promote well‐being for all at all ages. Target 3.4 of SDG3 specifically calls for a one‐third reduction in premature mortality from non‐communicable diseases, including tobacco‐related cancers, cardiovascular diseases and respiratory illness, by 2030. Dental professionals, given their unique position as oral health educators and their early detection of tobacco‐related oral manifestations, have a critical role to play in contributing to this global agenda. Training dental students in evidence‐based tobacco cessation counselling is therefore not merely a curricular objective but a meaningful contribution to the achievement of the SDG3 targets.

## 5. Conclusion

The prevalence of current smoking among the study participants was relatively low. Most current users were cigarette smokers and approximately two‐thirds reported smoking fewer than two cigarettes per day. Enforcement of policies should be followed strictly at all levels to prevent tobacco usage among students. Dental health professionals who smoke may not serve as effective role models for patients whom they counsel to quit tobacco use. As dentists are regarded as health educators, their involvement in tobacco consumption can create a negative impact on society. Therefore, it is imperative that dental students who are future dental professionals should develop adequate knowledge, attitudes and skills to address tobacco effectively, including training in patient counselling for tobacco cessation.

The Dental Council of India should place greater emphasis on tobacco education, and the dental curriculum should incorporate structured initiatives that encourage students to actively participate in comprehensive tobacco counselling services, in alignment with the Dental Council of India’s 2022 Reference Manual for Tobacco Cessation in Dental Settings [23], which provides a concrete framework for integrating cessation training into undergraduate dental programmes. Quitting tobacco is a cornerstone of public health efforts and is strongly linked to global goals of sustainable good health and well‐being (SDG3) because it leads to immediate and long‐term health improvements, reducing the burden of disease over a person’s lifetime.

## Author Contributions

**Deepak Kumar Singhal:** conceptualisation, methodology, project administration and resources, supervision, writing – review and editing. **Vathsala Patil:** investigation, writing – review and editing. **Nishu Singla:** data curation, formal analysis, writing – review and editing. **Ramprasad Vasthare Prabhakar:** supervision, validation, writing – review and editing. **Chetana Chandrashekar:** writing – review and editing, validation. **Jeevan V. Sambasivan:** data collection, formal analysis, writing – original draft.

## Funding

This study did not receive any specific funding.

## Disclosure

All authors have read and approved the final version of the manuscript. The corresponding author had full access to all of the data in this study and takes complete responsibility for the integrity of the data and the accuracy of the data analysis.

## Conflicts of Interest

The authors declare no conflicts of interest.

## Data Availability

The data supporting the findings of this study are not publicly available due to ethical and privacy considerations but may be made available from the corresponding author upon reasonable request.
